# Effect of Measurement System Configuration and Operating Conditions on 2D Material-Based Gas Sensor Sensitivity

**DOI:** 10.3390/nano13030573

**Published:** 2023-01-31

**Authors:** Jongwon Ryu, Seob Shim, Jeongin Song, Jaeseo Park, Ha Sul Kim, Seoung-Ki Lee, Jae Cheol Shin, Jihun Mun, Sang-Woo Kang

**Affiliations:** 1 Advanced Instrumentation Institute, Korea Research Institute of Standards and Science, Daejeon 34113, Republic of Korea; 2 Department of Physics, Chonnam National University, Gwangju 61186, Republic of Korea; 3 Precision Measurement, University of Science and Technology, Daejeon 34113, Republic of Korea; 4 School of Materials Science and Engineering, Pusan National University, Busan 46241, Republic of Korea; 5 Division of Electronics and Electrical Engineering, Dongguk University, Seoul 04620, Republic of Korea

**Keywords:** gas sensors, two-dimensional materials, measurement systems, test platforms, testbed

## Abstract

Gas sensors applied in real-time detection of toxic gas leakage, air pollution, and respiration patterns require a reliable test platform to evaluate their characteristics, such as sensitivity and detection limits. However, securing reliable characteristics of a gas sensor is difficult, owing to the structural difference between the gas sensor measurement platform and the difference in measurement methods. This study investigates the effect of measurement conditions and system configurations on the sensitivity of two-dimensional (2D) material-based gas sensors. Herein, we developed a testbed to evaluate the response characteristics of MoS_2_-based gas sensors under a NO_2_ gas flow, which allows variations in their system configurations. Additionally, we demonstrated that the distance between the gas inlet and the sensor and gas inlet orientation influences the sensor performance. As the distance to the 2D gas sensor surface decreased from 4 to 2 mm, the sensitivity of the sensor improved to 9.20%. Furthermore, when the gas inlet orientation was perpendicular to the gas sensor surface, the sensitivity of the sensor was the maximum (4.29%). To attain the optimum operating conditions of the MoS_2_-based gas sensor, the effects of measurement conditions, such as gas concentration and temperature, on the sensitivity of the gas sensor were investigated.

## 1. Introduction

Gas sensors are becoming increasingly important for gas leak detection, respiration measurement and analysis, and air quality monitoring in residential, industrial, medical, and environmental fields. There are various types of gas sensors, such as optical gas imaging, acoustic, and electrochemical. Optical gas imaging can detect hydrocarbon leak of the chemical plant in real-time from several meters away [1]. Acoustic gas sensor based on a compact microphone array can detect 0.1 mL/s leak via listening to the burst sound of invisible bubbles [2]. However, among all gas sensors, electrical or chemical resistance gas sensors are attracting the prime attention due to low cost and power consumption, small size, and simple fabrication process. To achieve cost-effective and high-performance electrochemical gas sensors with high sensitivity, selectivity, stability, and fast response-recovery time, different devices based on various active materials, such as silicon and its compounds [3,4,5,6], metal oxides [7,8,9,10], conductive polymers [11,12,13], carbon nanomaterials [14,15,16], and transition metal dichalcogenides (TMDCs) [8,17,18,19,20], have been extensively explored. To develop a high-performance gas sensor that satisfies the aforementioned demand characteristics, it is very important to select suitable sensor types for their working environments (temperature, gas species, etc.). Thus, establishing a measurement platform that can accurately evaluate the sensor performance according to the measurement conditions and system configuration is critical for successful device fabrication.

To evaluate the performance of the gas sensor, a testbed, an instrument that measures characteristics such as the sensitivity and detection limits under different operating conditions, is utilized in the early stages of development. The testbed for gas sensors is classified into two types of measurement systems: static and dynamic [9,21]. The static measurement system is a method of measuring in a confined state after injecting a test gas into a closed chamber, and the dynamic measurement system is a method of measuring by forming a continuous flow of test gas in a chamber with gas inlet and outlet. In a static measurement system [22,23,24,25], the gas sensor under test is placed in a sealed test chamber with a temperature control unit and a gas injection unit, and the whole gas test device is placed in a ventilation cabinet. During the sensor test, a preset amount of test gas was injected into the test chamber and when the resistance of the test sensor stabilized, the chamber was opened and the gas was let out and absorbed by the cabinet. The volume of the target gas injected according to the calculated concentration is always constant with respect to the fixed chamber volume. In a dynamic measurement system [26,27,28], also known as a flow-through system, a constant flow of the test gas is injected into the test chamber through the mass flow controllers (MFC). During the sensor test, the resistance variation of the test sensor inside the test chamber, in which the steady-state condition was established, was continuously recorded. Both systems require isolation valves and gas flow controllers to expose the gas sensor to the injected gas, a temperature controller to maintain the gas sensor at a constant temperature and a test chamber with a confined volume to avoid retarding the response of the sensor [9,21].

Accurate gas-sensing measurements require several considerations. The factors contributing to the measurement uncertainty include the purity of each gas, resolution of the MFC, temperature, and pressure deviation, which determine the concentration uncertainty of the mixed gas. In addition, to achieve accurate and reliable gas detection, it is necessary to reduce the measurement noise and time delay caused by various factors during performance evaluation. Among these factors, testbed configurations, such as the volume and shape of the test chamber, design of gas inlets, and gas-mixing units, should be carefully considered as these factors influence the time to a change in the test gas concentration [29,30,31,32,33]. Despite these considerations, there are few studies available on gas-sensing testbed configurations. Endres et al. [34] described the dependence of the gas flow regime on chamber configuration. Annanouch et al. [30] showed that the chamber dimensions influence the sensitivity of a gas sensor. Sedlák et al. [31,33] investigated the effect of the sensor position and rate and direction of gas flow on sensitivity. However, the implications of various system configurations of the apparatus for gas-sensing measurements, such as the gas inlet orientation to the sensor surface, the distance between the gas inlet and the gas sensor, and the angle between the gas inlets, have not been studied.

Herein, we developed a dynamic apparatus to evaluate the gas-sensing properties of MoS_2_-based gas sensors under a test gas flow, which allows simple adjustment of system configurations and low-noise measurements, and used this apparatus to better understand the system configuration dependence of the gas sensor performance. Through the evaluation platform developed in this study, the effect of the change in the position and angle of the gas inlet, which is a component of the chamber, on the gas sensitivity was investigated. To attain optimum working conditions of the MoS_2_ nanoflower-based gas sensor, we investigated the effect of measurement conditions such as temperature and gas concentration on the sensitivity of the 2D gas sensor. The sensor shows a linearly increasing response as the temperature and gas concentration increases. We demonstrate that the testbed is a traceability measurement platform where the system configuration and operating sequence do not affect the linear response of the gas sensor. The testbed we developed is not limited to only 2D material-based sensors, but will be applicable to measurement systems for various types of gas sensors.

## 2. Materials and Methods

### 2.1. Sensor Preparation

For the fabrication of the gas sensor based on MoS_2_ nanoflower, the MoS_2_, an active sensing layer, was grown on a p-type Si wafer with 300-nm-thick SiO_2_ (1–10 Ω·cm, iTASCO, Seoul, Korea) using metalorganic chemical vapor deposition (MOCVD) at a partial pressure ratio of 1.9 in the H_2_S to the Mo(CO)_6_ precursor [35]. Figure S1 (Supplementary Materials) shows the scanning electron microscopy (SEM) image of the MoS_2_ nanoflower sample, which is a sensing material of the gas sensor. The 2D MoS_2_ based sensing material has a unique flower shape, and is suitable for gas sensors due to its high surface-to-volume ratio. Furthermore, Figure S2 (Supplementary Materials) shows the Raman and photoluminescence (PL) spectra of the MoS_2_ nanoflower. The Raman spectra exhibits two peaks related to in-plane (E^1^_2g_, at 383.10 cm^−1^) and out-of-plane (A_1g_, 408.07 cm^−1^) vibration modes. The PL peak of the sample was detected at 1.90 eV, which corresponds to the A excitons. The bonding characteristic of the MoS_2_ nanoflower was captured by X-ray photoelectron spectroscopy (XPS), and the representative result is shown in Figure S3 (Supplementary Materials). As shown in Figure S3, the XPS survey spectrum located at 162.08, 229.08, 284.08, and 532.08 eV correspond to the S 2p, Mo 3d, C 1s, and O 1s, respectively, suggesting the existence of S, Mo, C, and O elements. To use the MoS_2_ as the active material of the gas sensor, MoS_2_ grown wafer was cut into 6 mm × 9 mm size and the unnecessary part of MoS_2_ film was removed by the scotch-tape method. As previously reported [36], a MoS_2_-based gas sensor was fabricated by depositing Au/Pt/Ti (150/50/50 nm) electrodes on the MOCVD-grown MoS_2_ sensing layer using a stencil mask and e-beam evaporator under high-vacuum conditions (<10^−4^ Pa). The gap between the electrodes was 100 µm and the open area of the gas sensor was 30 mm^2^. The gas sensor was attached to a printed circuit board with adhesive tape, and the electrical connection between the electrodes of the gas sensor and the tip of the printed circuit board was made using gold wire bonding. Figure S4 (Supplementary Materials) shows optical images of the actual gas sensor and Table S1 (Supplementary Materials) shows the basic parameters of the gas sensor fabrication.

### 2.2. Materials Characterization

Morphological analysis was performed using SEM (S-4800, Hitachi, Tokyo, Japan). The Raman and PL measurement were performed using a confocal Raman spectroscope (inVia, Renishaw, WUE, UK) with a 488-nm laser. The bonding characteristic was measured using XPS (Sigma Probe, Thermo VG Scientific, MA, USA).

### 2.3. Experimental Setup

**Figure 1:**
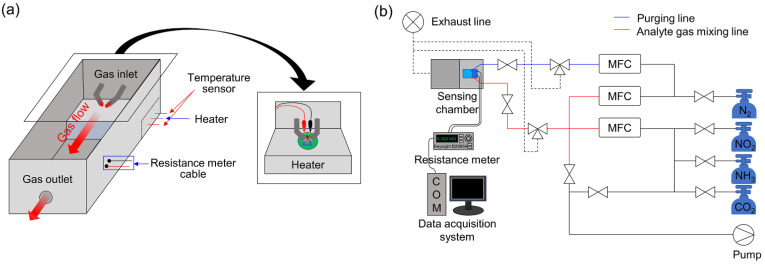
Schematic of a dynamic gas-sensing testbed developed in this work: (**a**) Gas-sensing test chamber with heating stage and electrical connection. (**b**) Overall system configuration showing the analyte and purge gas flow direction.

The sensing test chamber was made of aluminum and had a volume of 0.01 m^3^. The test chamber was composed of a two-gas inlet, a gas outlet, two cartridge heaters, and an electrical feedthrough to control resistance meter, as shown in Figure 1a. Two Incoloy cartridge heaters with 13 mm outer diameter and 100 mm sheath lengths were inserted into drilled holes inside the sample stage within the test chamber to control the temperature of the gas sensor. The test chamber had two flexible gas inlet tubes (a test gas inlet and a purge gas inlet) and one gas outlet, which were facing each other. This gas inlet/outlet configuration allowed a gas mixture with a predetermined concentration to flow inside the test chamber at a constant flow rate. The flexible gas inlet tube enabled the distance between the gas inlet and the 2D gas sensor, and the gas inlet orientation toward the sensor to be altered. The gas sensors mounted on the sample stage inside the test chamber were connected to the resistance meter (B2985A, Keysight, SR, USA) using SMA connectors to measure and record the electrical resistance of the sensor before and after exposure to the test gas. The variation in the sensor resistance over time was measured using a computer-controlled measurement and data acquisition system, in which the gas valve operation and gas flow rate were controlled by LabVIEW-based software (LabVIEW 14.0, NI, Texas, USA).

Figure 1b shows overall system configuration and the typical procedure of the gas-sensing test was as follows. After the fabricated gas sensor was placed onto the sample stage inside the test chamber, the test device was heated at a certain moderate temperature until its resistance stabilized. N_2_ gas was then introduced into the test chamber by a MFC through the gas inlets to obtain N_2_ saturated condition of the gas sensor. Once the saturation condition was set, a DC voltage of +1 V was applied to the sensor. Subsequently, the sensor was exposed to the analyte gas such as NO_2_, NH_3_, and CO_2_ gas (diluted with N_2_ balance gas) at the desired concentration for 300 s. The analyte gases are the certified reference material manufactured by the Korea Research Institute of Standards and Sciences. The total gas flow rate was maintained at 100 sccm, and the variation in the sensor resistance was measured. After each successive test, the chamber was evacuated and purged with N_2_ gas to remove residual gases and recover the sensor. Finally, the sensitivity of the gas sensor was calculated from the resulting changes in sensor resistance.

## 3. Results and Discussion

In this study, the change in sensitivity of the gas sensor was investigated using nitrogen dioxide gas as the test gas. The sensitivity [37,38,39] of the gas sensor was defined using Equation (1):

(1) $$\mathrm{Sensitivity}\;\left(\%\right)=\frac{\left|\Delta R\right|}{R_{0}}=\frac{\left|R_{g}-R_{0}\right|}{R_{0}}\;\left(\%\right),$$

where R_g_ represents the sensor resistance to the analyte gas and R_0_ represents the initial sensor resistance in a N_2_ atmosphere.

### 3.1. Effect of the System Configuration and Operating Sequence on Sensor Signal

**Figure 2:**
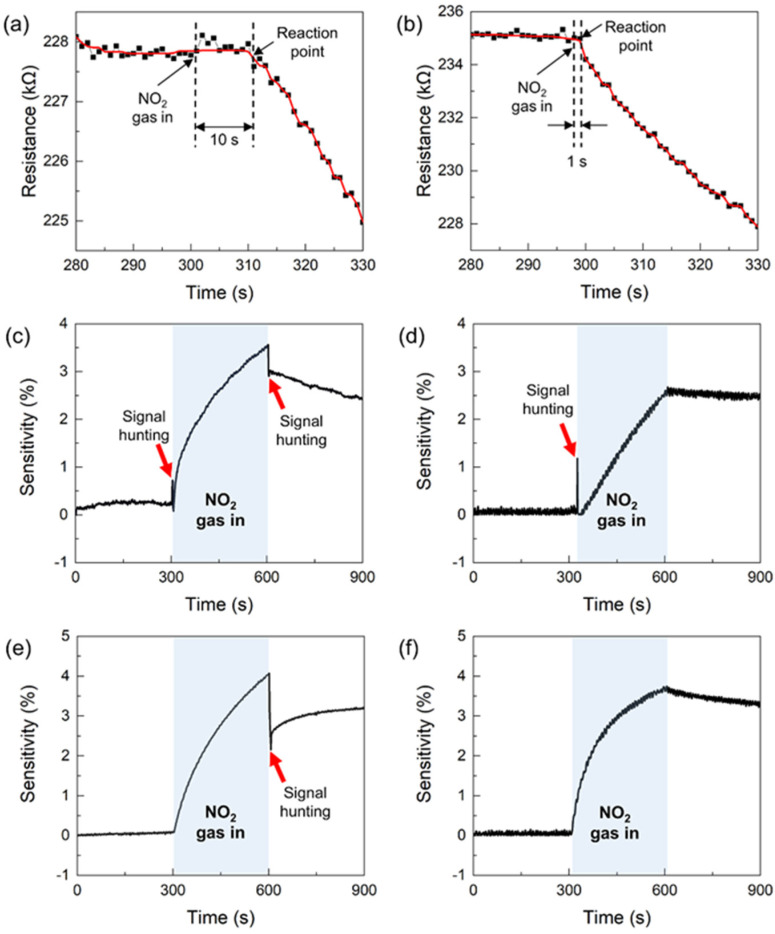
Changes in resistance of the MoS_2_ gas sensor exposed to 5 ppm of NO_2_. (**a**) Single gas inlet (common operating sequence); delayed reaction at the point of NO_2_ exposure. (**b**) Two-gas inlets (time delay on/off switching of gas inlet valve); no delayed reaction. (**c**) No time delay control (common operating sequence). (**d**) One-second time delay control applied to the recovery step. (**e**) One-second time delay control applied to the NO_2_ exposure step. (**f**) One-second time delay control applied to both the NO_2_ exposure and the recovery step.

In many gas-sensor studies, most gas-sensing measurement systems usually contain a single gas inlet, which influences the gas sensor performance [40,41,42,43,44]; when the purge gas is introduced, the remaining test gas may be unintentionally introduced from the gas-mixing part. Therefore, a single gas inlet system may cause unexpected abnormal signals behavior, called signal hunting, and the delayed reaction of the gas sensor. Figure 2a shows the delayed reaction of the sensor at the point of NO_2_ exposure obtained from the gas-sensing test carried out using a single gas inlet system. The sensor showed a resistance change 10 s after exposure to NO_2_ gas. This is a result of the residence time required for NO_2_ gas to travel from the MFC outlet to the sensor. Conversely, Figure 2b shows the immediate response of the sensor at the same point by removing the signal hunting using a two-gas inlet system; the mixed test gas and purge gas were separately introduced into the chamber through two-gas inlets. With regard to a system with a single gas inlet, it is possible to evaluate the effects by changing the flow rate of each MFC in advance or by adding a valve in front of each MFC to adjust the on/off duration in advance based on the gas transfer time. However, these alternatives may complicate the system configuration and operating sequence. Consequently, we simplified and improved the system configuration by applying two-gas inlets.

Further experimental study on how the system configuration and operating sequence influence the sensor signal was conducted. Figure 2c shows the transient response of the MoS_2_ gas sensor to a NO_2_ concentration of 5 ppm, which was the result obtained from the gas-sensing test conducted in the two-gas inlets system. When the on/off control of the MFC and gas valve was simultaneously operated at each measurement step, hunting signals occurred at the point of NO_2_ exposure and N_2_ purge. These hunting signals appear because if each valve is opened and closed simultaneously, the gas supply is momentarily stopped while the gas moves from the valve close to the gas inlet into the chamber. To remove unexpected hunting behaviors, the on/off switching time control of the gas inlet valve, that is, one-second time delay, was controlled. Figure 2d,e show the sensor responses obtained from the gas-sensing tests conducted in a time-delay-controlled operating sequence. When the time delay control was applied to the last recovery step (the exposure valve closed 1 s after the purge valve opened), signal hunting did not occur at the point of the N_2_ purge, as shown in Figure 2d. In contrast, when time delay control was applied to the exposure step (the purge valve closed 1 s after the exposure valve opened), signal hunting did not occur at the point of NO_2_ exposure, as shown in Figure 2e. Consequently, when the time delay control was applied to both the exposure and purge steps, no signal hunting appeared at the point of NO_2_ exposure and N_2_ purge, as shown in Figure 2f.

### 3.2. Effect of the Gas Inlet Orientation and Distance to Sensor Surface on Sensitivity

**Figure 3:**
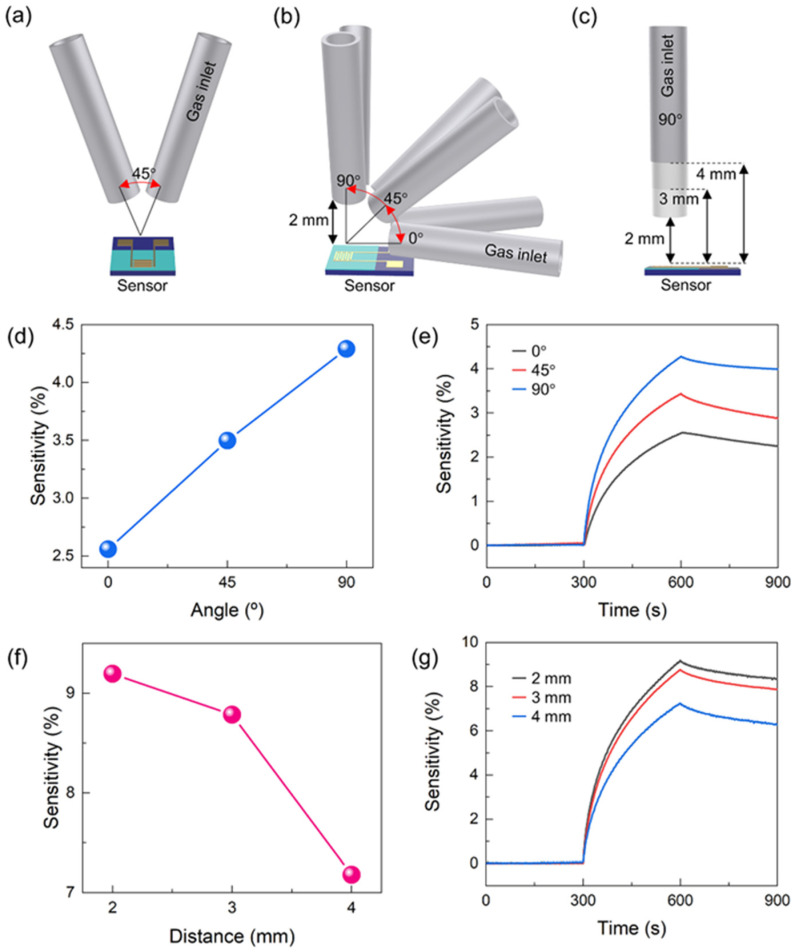
Schematic representation of gas inlet-gas sensor geometry showing (**a**) the angle between the two-gas inlets (front view), (**b**) the angle, and (**c**) the distance between the gas inlet and the sensor (side view). The sensing responses of the MoS_2_ gas sensor to 5 ppm of the analyte NO_2_ (**d**,**e**) at different angles between the gas inlet and the sensor (0°, 45°, and 90°) and (**f**,**g**) at different distances between the gas inlet and the sensor (2, 3, and 4 mm) at the gas inlet angle of 90° under room temperature (21 °C).

To investigate the influence of different gas inlet orientations toward the sensor and distance to the sensor on sensitivity, the MoS_2_ gas sensors were exposed to 5 ppm of the analyte NO_2_ gas injected at various angles and distances between the gas inlet and the sensor surface. During all comparison experiments, the angle between two-gas inlets was maintained at 45°, as shown in Figure 3a. In the gas inlet orientation experiment, the distance between the gas inlet and the sensor surface was maintained at 2 mm and the effects of the three different angles of 0°, 45°, and 90° were evaluated as shown in Figure 3b. The 90° angle position means that the gas flow direction was perpendicular to the sensor surface. The distance comparison experiment was performed by changing the distance between the gas inlet and the sensor surface to three different distances of 2, 3, and 4 mm at 90° angle position, as shown in Figure 3c. Their sensitivity was measured using the two-gas inlet system and time delay on/off switching of the gas inlet valve at room temperature (21 °C) and atmospheric pressure (1 atm). As the vertical angle increased from 0° to 90°, the sensor sensitivity linearly increased, as shown in Figure 3d,e, with 2.6%, 3.5%, and 4.3% for angles 0°, 45°, and 90°, respectively. It can be seen that the impingement rate of the analyte gas molecule on the sensor surface, the number of gas molecules that collide with the surface per second, and the unit area increased with the gas inlet angle (from 0° to 90°).

Figure 3f,g show the changes in the sensor sensitivity with the distance between the gas inlet and the sensor surface. As the gas inlet to the sensor distance increased from 2 to 4 mm, the sensor sensitivity gradually decreased to 9.2%, 8.8%, and 7.8%, respectively. These results can be explained by the aforementioned description of the changes in the impingement rate of the NO_2_ molecule on the sensor surface, as with the effect of the gas inlet angle on the sensor sensitivity. As the gas inlet moves farther away from the sensor surface, interference with the analyte gas transfer to the surface by atmospheric gas occurs more frequently during its travel. This is due to its extremely limited mean free path under atmospheric pressure, which causes a decrease in the impingement rate of analyte gas molecules. Table S2 (Supplementary Materials) shows the normalized gas sensitivity of the MoS_2_ gas sensor to 5 ppm of the analyte NO_2_ with different angles and distances between the gas inlet and the sensor surface.

### 3.3. Effect of Gas Concentration and Temperature on Sensitivity

**Figure 4:**
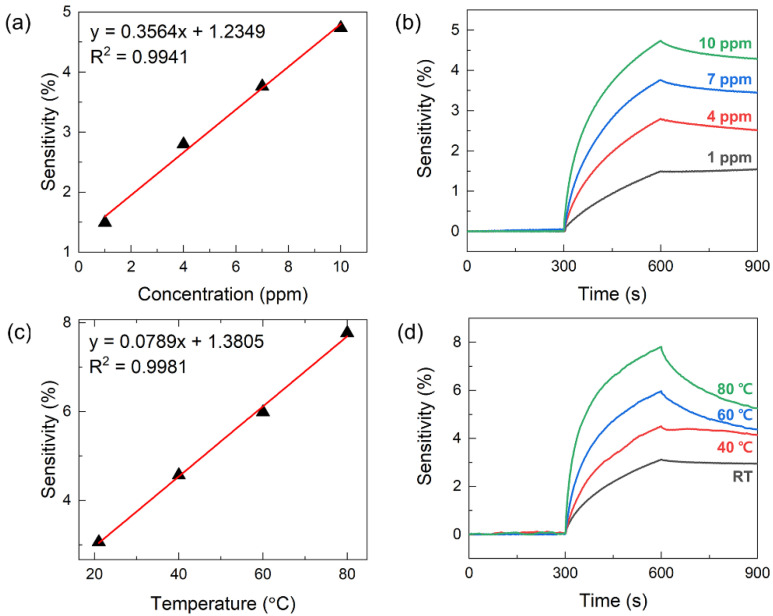
(**a**,**b**) The sensing responses of the MoS_2_ gas sensor to different analyte NO_2_ concentrations ranging from 1 to 10 ppm obtained from the two-gas inlet system with optimal configuration (the angle of 90° and the distance of 2 mm) under room temperature. (**c**,**d**) The sensing responses of the MoS_2_ gas sensor to 5 ppm of the analyte NO_2_ at different operating temperatures obtained from the two-gas inlet system with optimal configuration (the angle of 90° and the distance of 2 mm) under room temperature.

We further investigated the analyte gas concentration and temperature dependence of the sensor sensitivity to determine the optimum operating conditions of the MoS_2_-based gas sensor. The gas-sensing responses of the sensor for various NO_2_ concentrations from 1 to 10 ppm are depicted in Figure 4a,b, showing an increase in the sensor sensitivity with increasing NO_2_ concentration. The sensor responses were obtained from the two-gas inlet system with optimal configuration (angle of 90° and distance of 2 mm) using the time delay on/off valve control. The gas concentration was determined by the analyte to balance the gas volume ratio under standard atmospheric pressure. As the NO_2_ concentration increased from 1 to 10 ppm, the sensor sensitivity increased linearly to 1.5%, 2.8%, 3.8%, and 4.8% at concentrations 1, 4, 7, and 10 ppm, respectively.

Figure 4c,d show the gas-sensing responses of the sensor to 5 ppm NO_2_ at different operating temperatures obtained from the two-gas inlet system with a gas inlet angle of 90° and a gas inlet to sensor distance of 2 mm at room temperature. As the temperature increased from 21 to 80 °C, the sensitivity increased linearly to 3.1%, 4.6%, 6.0%, and 7.8% for temperatures of 21, 40, 60, and 80 °C, respectively. The MoS_2_ nanoflower layer of the 2D gas sensor used in this study acts as a p-type semiconductor, which is attributed to a decrease in its resistance upon exposure to NO_2_ gas. When NO_2_ acts as the oxidizing gas adsorbed on the surface of the MoS_2_ active layer, the influence of the low-resistance hole accumulation region on the overall change in the sensor resistance is relatively more dominant than that of the high-resistance electron shell depletion region, and the total resistance of MoS_2_ decreases significantly [10,45,46]. The semiconductor gas sensor further promotes the aforementioned change in resistance at high temperature the charge transfer between the adsorbed gas species and the sensing surface is improved as the temperature increases.

## 4. Conclusions

In summary, we developed a dynamic gas sensor test platform that allows simple adjustments of the measurement system configurations and low-noise measurement. This apparatus allows us to better understand how the measurement system configuration and operating conditions affect the sensing response of the MOCVD-grown MoS_2_ gas sensor. By using a two-gas inlet system and time delay on/off control of the gas inlet valve, abnormal signal hunting was removed, leading to the enhancement of the response time without delayed reaction. It was found that the variations in the system configuration (the gas inlet orientation and the gas inlet to sensor distance) significantly affected the sensor sensitivity. The sensor sensitivity was enhanced by increasing the gas inlet angle (gas flow direction perpendicular to the sensor surface) and decreasing the distance from the sensor. Furthermore, the analyte gas concentration and temperature dependence of the sensor sensitivity were investigated, and it was confirmed that the higher the concentration and temperature, the linearly higher the sensitivity of the gas sensor. For more precise and reliable gas-sensing measurements, further discussion on the critical factors (analyte gas species, gas flow rate, test chamber volume, surface area of the sensor, etc.) in addition to the measurement system configuration influencing the sensor responses, signal noise, and cross-interference, should be conducted.

## Data Availability

The data presented in this study are available on request from the corresponding author.

## Supplementary Materials

The following supporting information can be downloaded at: https://www.mdpi.com/article/10.3390/nano13030573/s1, Figure S1: SEM image of the MOCVD grown MoS_2_ nanoflower.; Figure S2: Raman and PL spectra of the MoS_2_ nanoflower.; Figure S3: XPS survey spectrum of MoS_2_ nanoflower.; Figure S4: Optical image of the gas sensor attached to a printed circuit board and the connected electrodes using gold wire bonding.; Table S1: Basic parameters of the gas sensor fabrication.; Table S2: Normalized gas sensitivity of the MoS_2_ gas sensor to 5 ppm of the analyte NO_2_ with different angles and distances between the gas inlet and the sensor surface.
