# Supplementary material for: Effect of Measurement System Configuration and Operating Conditions on 2D Material-Based Gas Sensor Sensitivity
Source: Nanomaterials (Basel). 2023 Jan 31;13(3):573. doi: 10.3390/nano13030573 (PMC9919673; doi:10.3390/nano13030573)
Supplement: Supplementary file 1 [file nanomaterials-13-00573-s001.zip › nanomaterials-2159554_Supplementary materials-done.pdf]

nanomaterials

Supplementary Materials

Effect of Measurement System Configuration and Operating Conditions on 2D Material-Based Gas Sensor Sensitivity

Jongwon Ryu [1,†], Seob Shim [1,†], Jeongin Song [1,2], Jaeseo Park [1,3], Ha Sul Kim [2], Seoung-Ki Lee [4], Jae Cheol Shin [5], Jihun Mun [1,*] and Sang-Woo Kang [1,3,*]

1 Advanced Instrumentation Institute, Korea Research Institute of Standards and Science, Daejeon 34113, Republic of Korea
2 Department of Physics, Chonnam National University, Gwangju 61186, Republic of Korea
3 Precision Measurement, University of Science and Technology, Daejeon 34113, Republic of Korea
4 School of Materials Science and Engineering, Pusan National University, Busan 46241, Republic of Korea
5 Division of Electronics and Electrical Engineering, Dongguk University, Seoul 04620, Republic of Korea
* Correspondence: jmun@kriss.re.kr (J.M.) ; swkang@kriss.re.kr (S.W.K.)
† These authors contributed equally to this work.

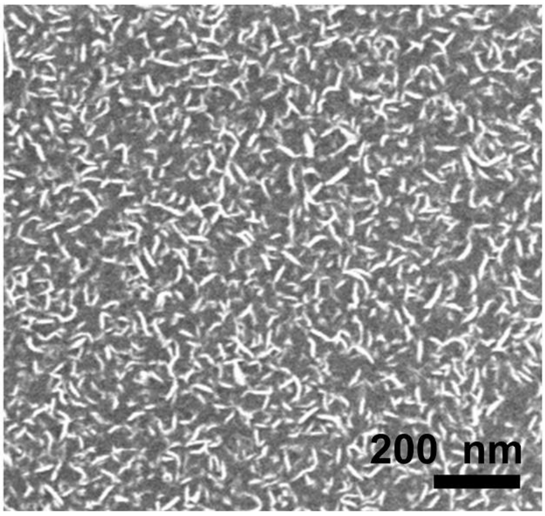

Figure S1. SEM image of the MOCVD grown MoS_2 nanoflower.

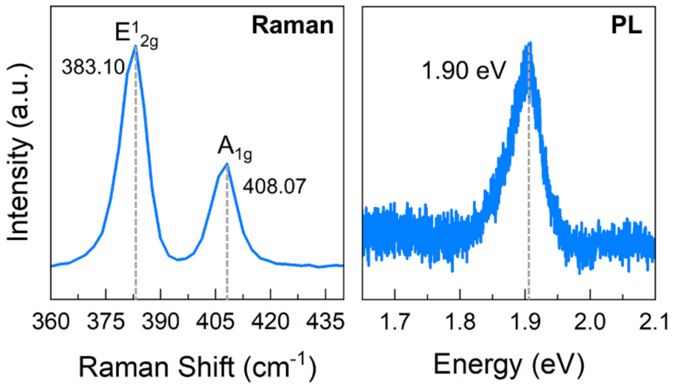

Figure S2. Raman (left) and PL (right) spectra of the MoS_2 nanoflower.

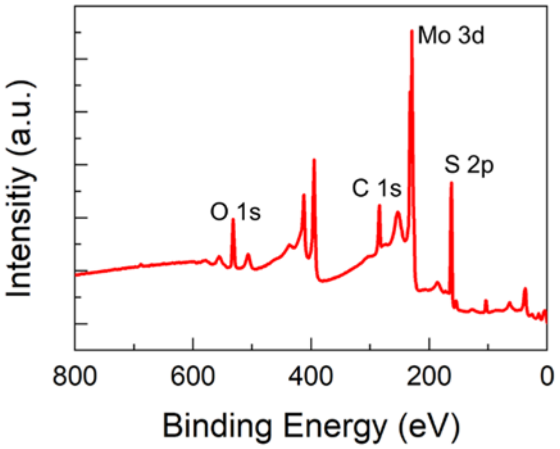

Figure S3. XPS survey spectrum of MoS_2 nanoflower.

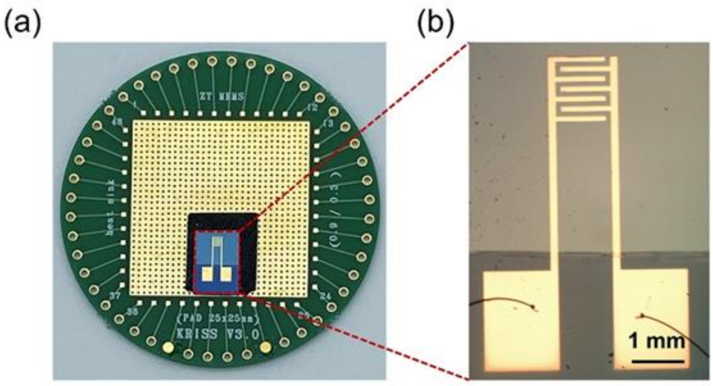

Figure S4. (a) Optical image of the gas sensor attached to a printed circuit board and the connected electrodes using gold wire bonding. (b) Optical microscope image of the gas sensor.

Table S1. Basic parameters of the gas sensor fabrication.

Sensor dimension	Channel dimension	Electrode thickness	Electrodes gap
6 mm × 9 mm	6 mm × 5 mm	250 nm	100 μm

Table S2. Normalized gas sensitivity of the MoS_2 gas sensor to 5 ppm of the analyte NO_2 with different angles and distances between the gas inlet and the sensor surface.

Angle (°)	Distance (mm)	Normalized sensitivity (%)
0	2	0.6
45	2	0.8
90	2	1
90	3	0.9
90	4	0.8
